# Field trials reveal the complexities of deploying and evaluating the impacts of yeast-baited ovitraps on *Aedes* mosquito densities in Trinidad, West Indies

**DOI:** 10.1038/s41598-022-07910-0

**Published:** 2022-03-08

**Authors:** Lester D. James, Nikhella Winter, Akilah T. M. Stewart, Rachel Shui Feng, Naresh Nandram, Azad Mohammed, Molly Duman-Scheel, Ethan Romero-Severson, David W. Severson

**Affiliations:** 1 Department of Life Sciences, Faculty of Science & Technology, The University of the West Indies, St. Augustine Campus, St. Augustine, Trinidad and Tobago; 2 Insect Vector Control Division, Ministry of Health, St. Augustine, Trinidad and Tobago; 3 Theoretical Biology and Biophysics Group, Los Alamos National Laboratory, Los Alamos, NM USA; 4 Department of Medical and Molecular Genetics, Indiana University School of Medicine, South Bend, IN USA; 5 Department of Biological Sciences and Eck Institute for Global Health, University of Notre Dame, Notre Dame, IN USA

**Keywords:** Urban ecology, Biotechnology, Diseases

## Abstract

The use of lure-and-kill, large-volume ovitraps to control *Aedes aegypti* and *Aedes albopictus* populations has shown promise across multiple designs that target gravid females (adulticidal) or larvae post-oviposition (larvicidal). Here we report on a pilot trial to deploy 10 L yeast-baited ovitraps at select sites in Curepe, Trinidad, West Indies during July to December, 2019. Oviposition rates among ovitraps placed in three Treatment sites were compared to a limited number of traps placed in three Control areas (no *Aedes* management performed), and three Vector areas (subjected to standard Ministry of Health, Insect Vector Control efforts). Our goal was to gain baseline information on efforts to saturate the Treatment sites with ovitraps within 20–25 m of each other and compare oviposition rates at these sites with background oviposition rates in Control and Vector Areas. Although yeast-baited ovitraps were highly attractive to gravid Aedes females, a primary limitation encountered within the Treatment sites was the inability to gain access to residential compounds for trap placement, primarily due to residents being absent during the day. This severely limited our intent to saturate these areas with ovitraps, indicating that future studies must include plans to account for these inaccessible zones during trap placement.

## Introduction

*Aedes aegypti* (L.) and to a lesser extent *Aedes albopictus* (Skuse) are the primary global mosquito vectors of multiple arboviruses, such as dengue, chikungunya, Zika, and yellow fever that significantly impact human health^1–4^. Efforts to prevent transmission or provide treatment for these arbovirus diseases by development of effective vaccines or antiviral drugs remain largely ineffective^5–8^. Disease prevention or reduction, globally, of these arboviruses has historically depended on efforts directed at controlling the mosquito vectors, most often by breeding site reduction and insecticide applications^9^. Notably resistance to all commonly used chemical insecticides is widespread among *Aedes* populations, thus limiting their effectiveness^10^. Therefore, a range of alternative methods that target larval or adult stages are being developed and tested^11,12^.

Gravid *Aedes* females actively seek to oviposit in man-made containers in and around human dwellings^13,14^, with a majority of mosquito breeding often seen in neighborhoods with low and medium socioeconomic levels^15^. The unreliability of piped water services in these areas, encourages active water storage in drums and tanks, as well as discarded container availability around houses, which combine to facilitate mosquito breeding^16,17^. Gravid *Aedes* females are known to practice ‘skip oviposition’, wherein they prefer to distribute eggs across multiple containers if given a choice, thereby maximizing the numbers of larval positive containers that mosquito control programs need to target^18,19^. Still, uncovered water storage drums and tanks are often the most prolific sources of *Aedes* mosquitoes in neighborhoods^14,19–22^.

Ovitraps consisting of small containers (usually ~ 500 ml) with water and an oviposition substrate have been employed as *Aedes* surveillance tools for decades^23–25^. More recently, concerted efforts have been directed at developing lethal ovitraps that: (1) are larger volume, (2) often spiked with attractants to lure gravid females, and (3) may include physical mechanisms like sticky boards to capture females that enter the traps^26^. The two most advanced lethal ovitraps are the Gravid *Aedes* Trap (GAT) and the *Aedes* Gravid Ovitrap (AGO). The GAT has a 10 L volume and contains 3 L of water, with lethality typically provided by an insecticide surface spray^27^. The AGO has a 19 L volume and contains 10 L of water, with lethality provided by a sticky surface adhesive^28^. Both traps utilize a hay infusion for enhanced attraction of gravid females. These larger volume traps have been shown to out-compete smaller volume lethal ovitrap designs^26^.

Large volume lethal ovitraps, while effective in reducing mosquito populations, face challenges relative to insecticide resistance as well as adverse effects on non-target organisms. There is a critical need to develop biofriendly pesticides that can easily be integrated into lethal ovitrap control programs. A promising technology, the RNA interference (RNAi) pathway is active in most eukaryotic cells and has evolved to silence gene expression with high specificity by the production of small interfering RNAs (siRNAs) of short length (21–25 bp)^29^. This pathway can be manipulated for the biofriendly control of mosquitoes that transmit pathogens to a vertebrate host by sequence-specific design of siRNAs that target critical genes in the mosquito^30^. The potential for successful development of RNAi-based larvicides for mosquitoes and the biomanipulation of the yeast *Saccharomyces cerevisiae* to produce them as short hairpin RNAs (shRNAs) has been shown to be highly effective and specific in controlled laboratory studies^30,31^, as well as simulated field and semi-field trials^32,33^.

Our previous laboratory assays with small volume (500 ml) containers have shown that gravid *A. aegypti* females were significantly more likely to oviposit in those baited with yeast compared to those with water only^30^. Subsequent laboratory and semi-field studies in Indiana, USA, as well as small-scale field trials in Trinidad, West Indies, all conducted with large volume ovitraps (7.5 L or 10 L) confirmed a preference to oviposit in those baited with yeast by *A. aegypti* as well as *A. albopictus* females^34^. In this study, we conducted a preliminary, small-scale field trial in neighborhoods around Curepe, Trinidad. Our goal was to obtain information on household access for ovitrap placement, general attractiveness of inactive yeast-baited ovitraps to gravid *Aedes* females when placed in a natural urban environment, and numbers of eggs laid in individual traps throughout the typical breeding season. These results will be used to facilitate planning for large-scale field trials with active long-lasting yeast formulations currently being developed and tested in laboratory studies.

## Results

### Treatment block demographics

**Figure 1:**
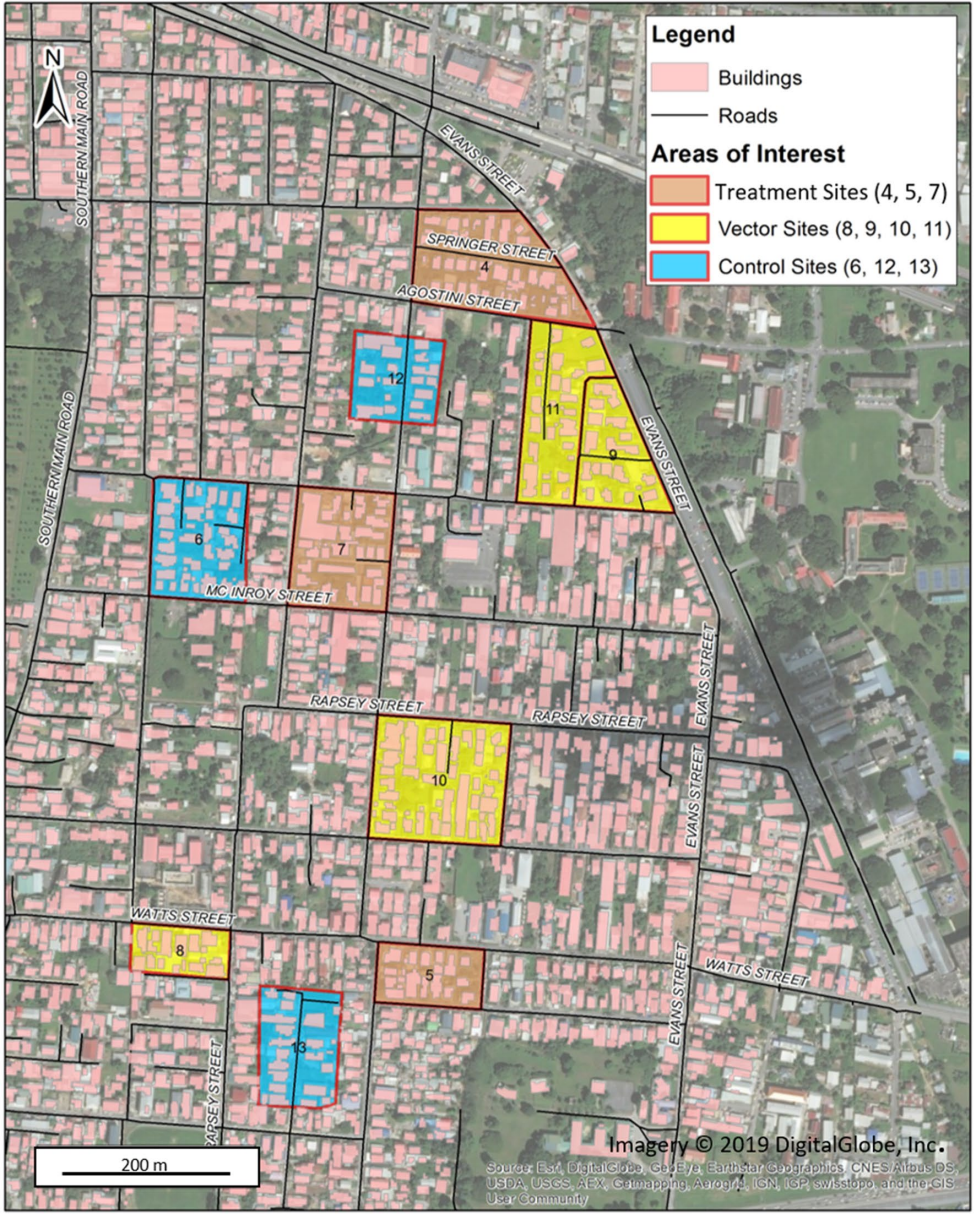
Map of study area in Curepe, Trinidad, West Indies from July to December 2019. Block areas identifying sites for Control, Vector, and Treatment yeast-baited 10 L ovitrap placement are highlighted. Figure was prepared with Environmental Systems Research Institute (ESRI). "Topographic basemap.” "World Topographic Map." (Sept. 28, 2021). https://www.arcgis.com/home/item.html?id=10df2279f9684e4a9f6a7f08febac2a9. (May 21, 2019).

The three selected Treatment blocks varied in total area from 9,588 to 22,558 m^2^ (Table 1) and were representative of most blocks in the Curepe study area (Fig. 1). The majority of buildings were private residences (73.7–85.7%), with some apartment complexes and a small number of commercial buildings. Nearly all private residences and apartment complexes were occupied, housing from 70 to 131 total people per block. A survey to identify all potential water-holding containers, irrespective of whether they held water at the time or not, within accessible household compounds that could represent sources for *Aedes* breeding prior to ovitrap distribution (May 27–29, 2019) identified a broad range of container types, including tanks that could be or were being used for water storage to a wide variety of miscellaneous small containers like flower pots and trays, plastic bottles, and metal cans. *Aedes* breeding activity observed in these containers was minimal, with only three positive containers observed among containers that were identified across all three Treatment blocks.

**Table 1:** Demographics for Treatment blocks in Curepe, Trinidad.

Block No.	4	5	7
Area (m^2^)	22,558	9588	17,360
Total No. buildings	36	19	28
Private residence	29	14	24
Apartment building	7	3	5
Commercial building	0	2	4
Total No. occupied	36	17	28
Total No. occupants	131	70	100
**Potential water containers**^a^ (%)			
Tanks	0/32 (18.9)	0/16 (10.4)	0/33 (6.6)
Drums	0/12 (7.1)	0/7 (4.5)	0/10 (2.0)
Tubs and Basins	2/65 (38.5)	0/55 (35.7)	1/107 (21.4)
Tires	0/2 (1.2)	0/5 (3.2)	0/17 (3.4)
Misc. Small	0/40 (23.7)	0/71 (46.1)	0/305 (61.0)
Indoors	0/18 (10.7)	0	0/28 (5.6)
Total	2/169	0/154	1/500

^a^No. Positive containers/Total containers inspected, May 27–29, 2019. All containers present were counted irrespective of whether they contained water or not. Data were limited to households that were accessible for container surveys.

### Vector block container surveys

Vector blocks were each surveyed for potential *Aedes* breeding containers five times during the study (except Block 8 that was not included in the initial June 27–28, 2019 survey) by IVCD staff (Table 2). Total container counts across survey dates for individual Vector blocks varied considerably due to limitations in accessing individual household areas as inhabitants were away when surveys were conducted. The most common container types based on the IVCD categories, were tubs and basins, and small miscellaneous. As observed in the Treatment blocks, *Aedes* breeding activity was minimal and mainly observed in the most common container types.

**Table 2:** Potential water containers and type for Vector blocks in Curepe, Trinidad, 2019.

Block number	Total units	Inspection date	No. units inspected	Tanks	Drums	Tubs and basins	Tires	Small Misc	Indoors	Total^a^
8	39	June 27–28	None							
		July 23	12	8	1/4	64	1	86	2	1/164
		September 3–4	18	7	7	165	0	147	17	0/343
		October 15	20	21	10	2/58	1/19	230	39	3/377
		November 20	21	5	2	2/79	2/21	122	15	4/244
9/11	79	June 27–28	38	43	4	99	5	139	9	0/299
		July 23	36	40	3	155	0	145	10	0/354
		September 3–4	40	42	9	1/173	0	170	19	1/411
		October 15	30	37	2	148	4	140	25	0/356
		November 20	42	49	2	180	12	241	43	0/518
10	82	June 27–28	26	35	9	184	6	273	4	0/424
		July 23	52	31	24	1/288	9	1/514	32	2/898
		September 3	34	35	10	2/119	5	1/295	32	3/496
		October 15	49	50	24	1/131	10	1/486	15	2/714
		November 20	54	28	2	2/83	2	170	15	2/284

^a^No. Positive containers/Total containers inspected.

### Ovitrap distribution and seasonal egg counts

**Figure 2:**
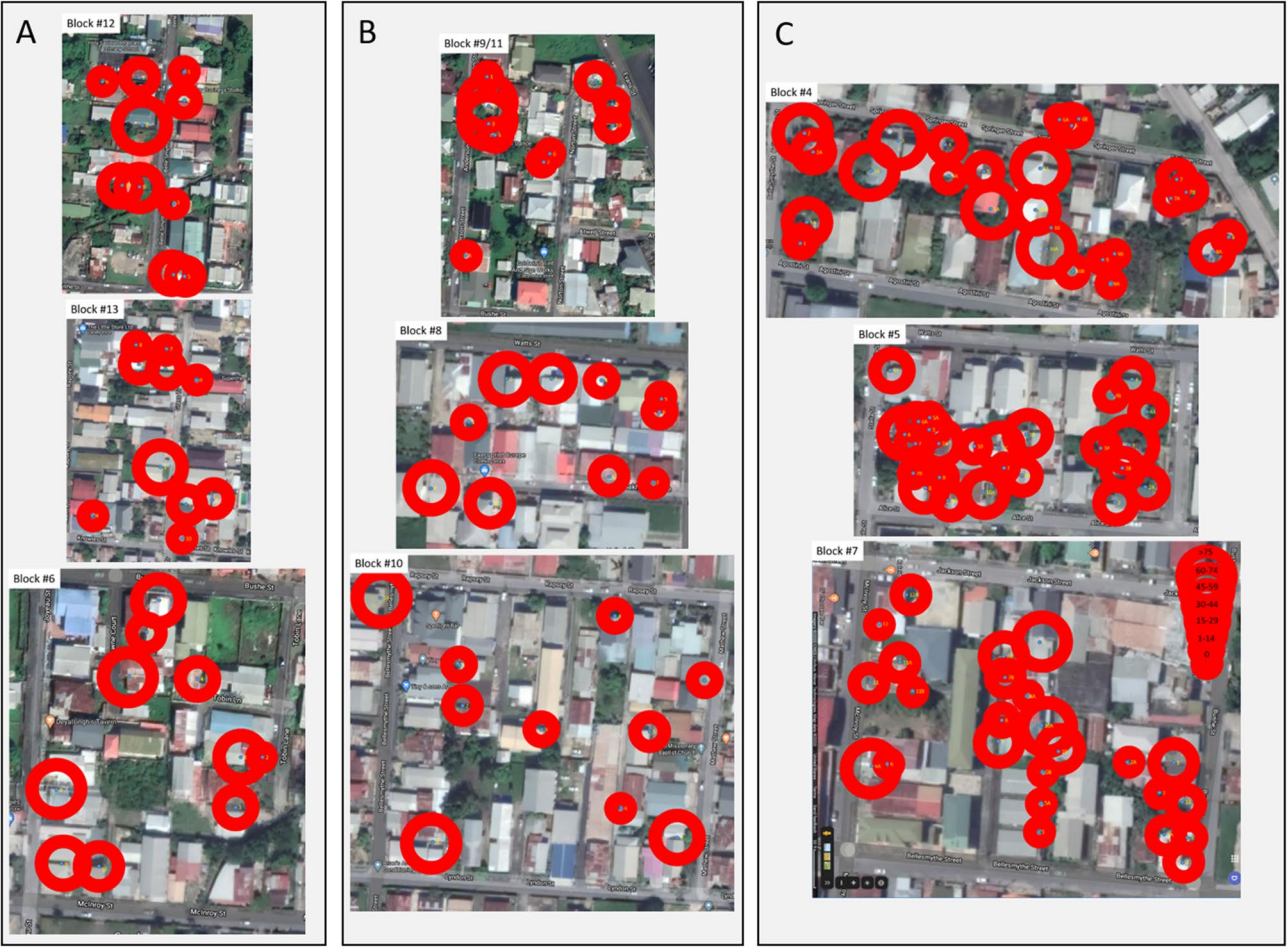
Individual yeast-baited 10 L ovitrap locations and median egg counts/trap from July to December 2019. (**A**) Control blocks; (**B**) Vector blocks; (**C**) Treatment blocks.

We collected data on *Aedes* oviposition in yeast-baited 10 L ovitraps for 23 weeks beginning June 26 and ending December 4. In addition to the 10 traps placed at each of the Control blocks (n = 3) and Vector blocks (n = 3), we were able to eventually place a total of 76 traps across the 3 Treatment blocks. One trap was removed midway during the study due to continued disturbance (block #4), leaving a total of 75 traps (n = 25 across each Treatment block). In addition, data were not collected for some weeks for some individual traps during the study due to disturbance by household pets, general household activities, flooding events, and owner absence. The complete data set is provided in Supplementary Table S1. Individual ovitrap locations and median egg counts/trap across the entire study period are shown for each block area in Fig. 2. Ovitrap placement across Treatment blocks was clearly nonrandom, primarily reflecting inaccessibility to some properties due to resident absence (presumably for work; only seven properties were inaccessible across all three Treatment blocks because the residents declined our request to place and monitor ovitraps on them), but also open mainly grassy areas and commercial buildings where ovitrap placement was unwarranted.

**Figure 3:**
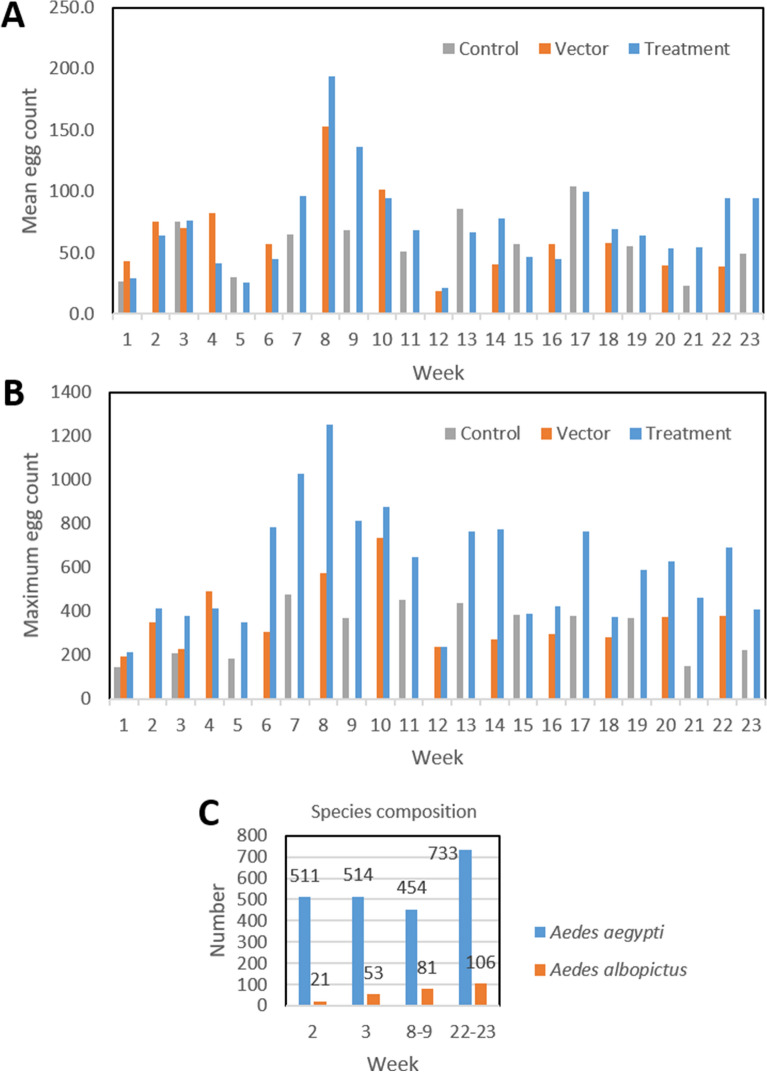
Summary of weekly egg collections of yeast-baited 10 L ovitraps positioned across Control, Vector, and Treatment blocks. (**A**) Mean egg counts; (**B**) Maximum egg counts; (**C**) *A. aegypti* vs. *A. albopictus* composition from eggs reared from select weekly samples.

During the course of the study, a total of 157,183 eggs were removed from the 135 ovitraps. Of these, 112,170 (71.4%) were collected from the 75 Treatment ovitraps and 45,013 (28.6%) from the 60 Control and Vector ovitraps. Mean weekly egg counts per ovitrap for the study period are shown in Fig. 3A, and ranged from ~ 20 to 200 eggs per trap, with considerable variability that generally reflects the typical rainy season in Trinidad that spans May to November^23,24^. Maximum egg counts per week per single ovitrap also showed considerable seasonal variability (Fig. 3B). The maximum egg count was n = 1251 during week 8 (August 20–21) in ovitrap #10 located in Treatment block #7. Of note, the individual ovitrap with the maximum weekly egg count was located within one of the Treatment blocks for 22 of the 23-week study period. This observation suggests that gravid *Aedes* females were attracted to these areas with more densely placed ovitraps. An evaluation of species composition based on periodic rearing of pooled eggs collected during weeks 2 (July 9–10), 3 (July 16–17), 8–9 (August 20–21, 27–28), and 22–23 (November 26–27, December 3–4) revealed a mixture of *A. aegypti* and *A. albopictus*, with *A. aegypti* being the predominant species (83.3–96.1%) throughout the study period (Fig. 3C). The inferred number of egg laying events showed much lower temporal variance and magnitude (Supplementary Fig. S1) compared to the raw data.

### Impact of increased density on index ovitraps in the Treatment block

**Figure 4:**
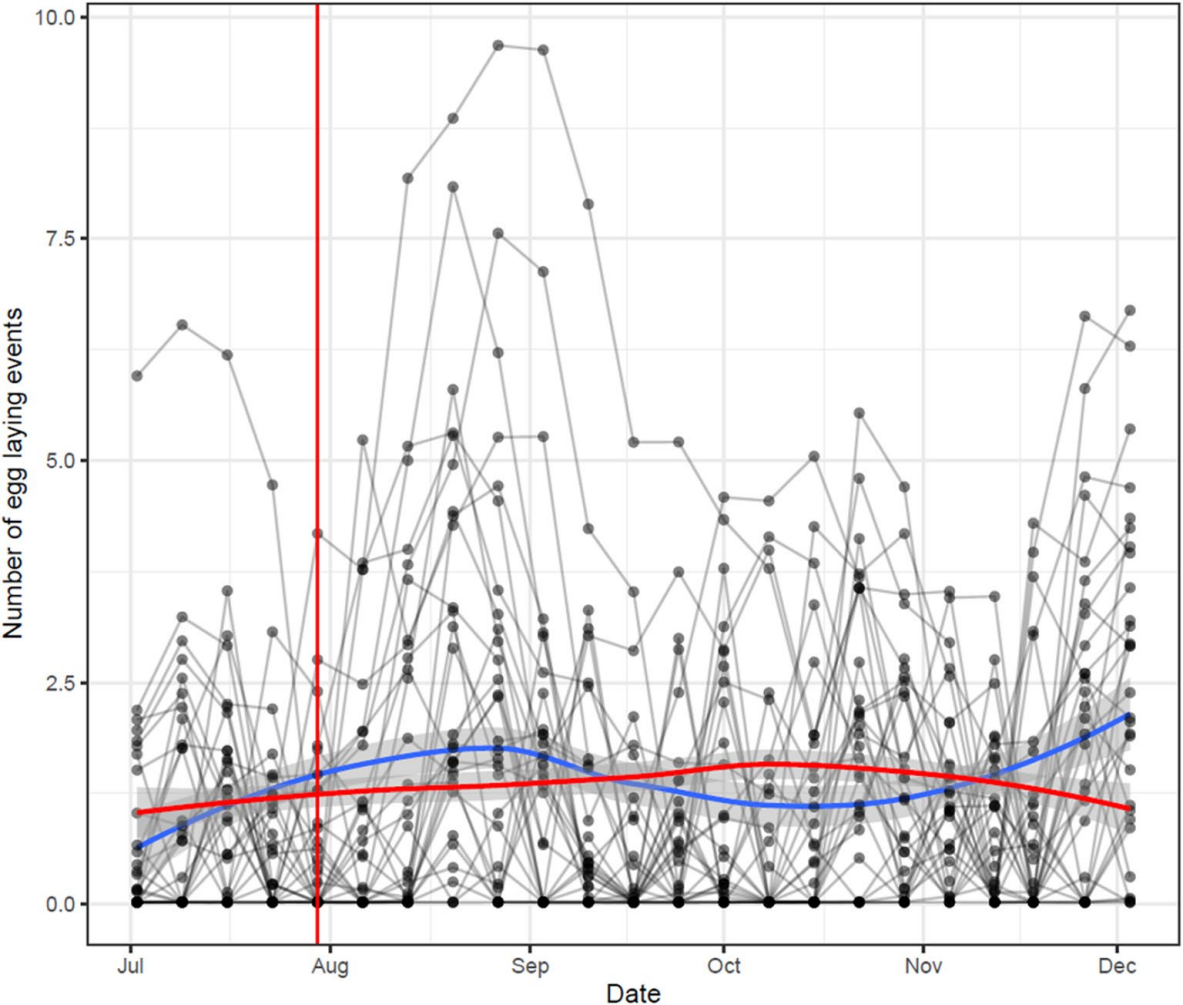
The inferred number of egg laying events in the index ovitraps in the Treatment blocks. The inferred number of egg-laying events over time for each index ovitrap in the Treatment blocks are plotted as lines. The index ovitraps were measured for 3 weeks before additional ovitraps were placed in the Treatment blocks (therefore increasing the number of removed eggs in those blocks). The red vertical line indicated the time at which new ovitraps were placed. The red and blue horizontal lines represent the loess curves for the index ovitraps in the Treatment block and the ovitraps in the combined control group (Control and Vector blocks), respectively.

The changes in index ovitraps in the Treatment block that were intended to be measured every week are shown in Fig. 4. During the third and fourth weeks, additional traps were added and measured (therefore increasing the number of eggs that were removed from the area) in the Treatment blocks. The increase in the density of traps in the Treatment block did not lead to a decrease in the number of egg laying events in the index traps. The small increase in the number of egg laying events after the third week may have been driven by the relatively small number of ovitraps that saw an uncharacteristically large increase in the number of egg laying events. The limited effect of increasing the density of ovitraps in the Treatment blocks is supported by the very similar temporal trend in the combined data for the Control and Vector blocks (Fig. 4).

### Impact of physical relationship between 10 L yeast-baited ovitraps and observed oviposition activity

**Figure 5:**
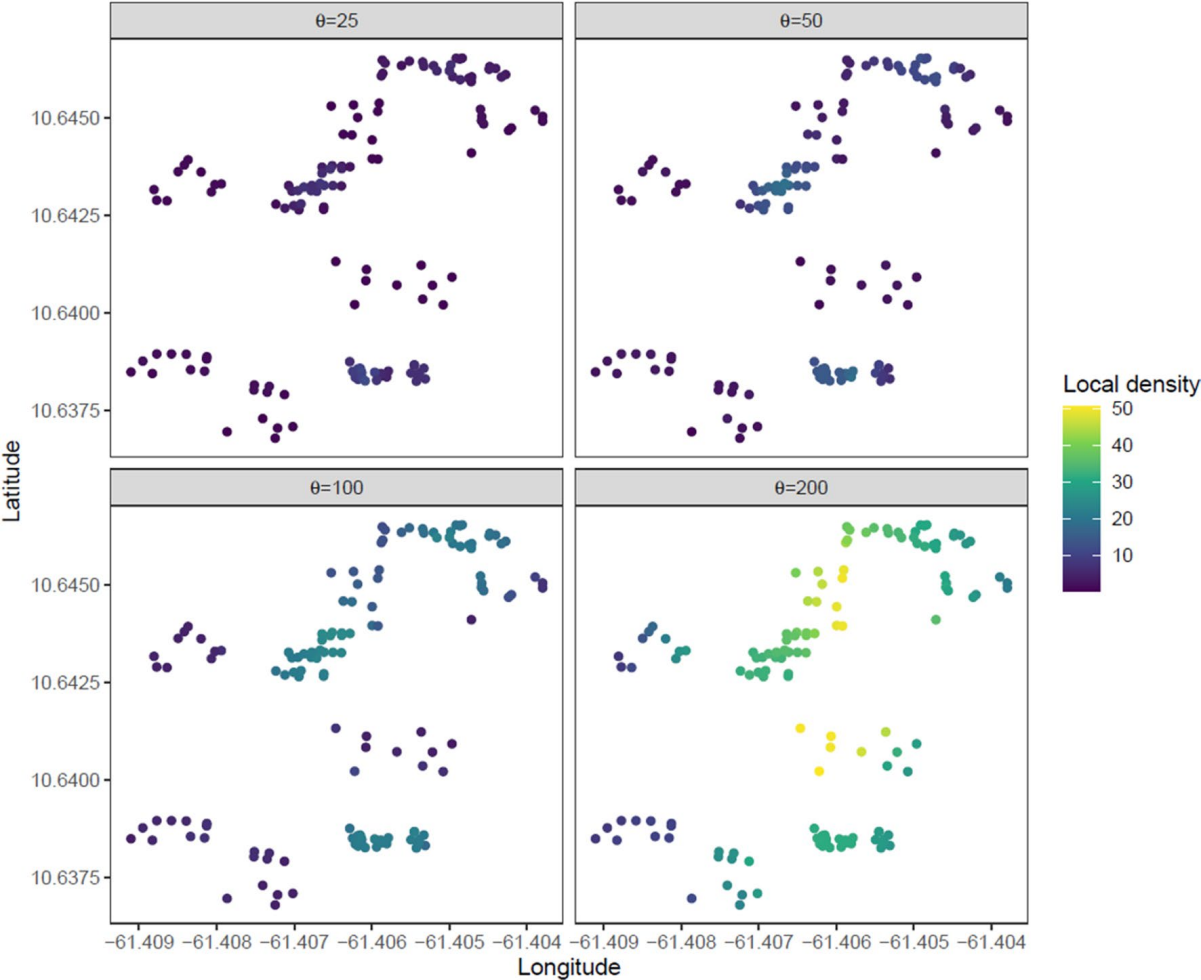
Relative positions of the ovitraps in space with illustration of local density measure. The relative physical locations of ovitraps are shown as dots with colors indicating the values of the local density measure under the four parameter levels used in the analysis. Heterogeneity in the spatial distribution of traps allows us to examine the effect of varying the local density of traps on the number of observed mosquito eggs.

To look more broadly for evidence of a local effect of ovitraps on the egg laying dynamics of the local mosquito population, we considered the local density of other ovitraps as a predictor of the number of egg laying events in an index ovitrap. The relative physical relationships of all 135 ovitraps is shown in Fig. 5, with the local density shown in color. As the ovitraps were not uniformly placed, they showed patterns of clustering which led to variance in the local density measures. No obvious differences were observed between egg laying events from the Control and Vector blocks (Supplementary Fig. S2), so data from both blocks were combined in this analysis.

**Figure 6:**
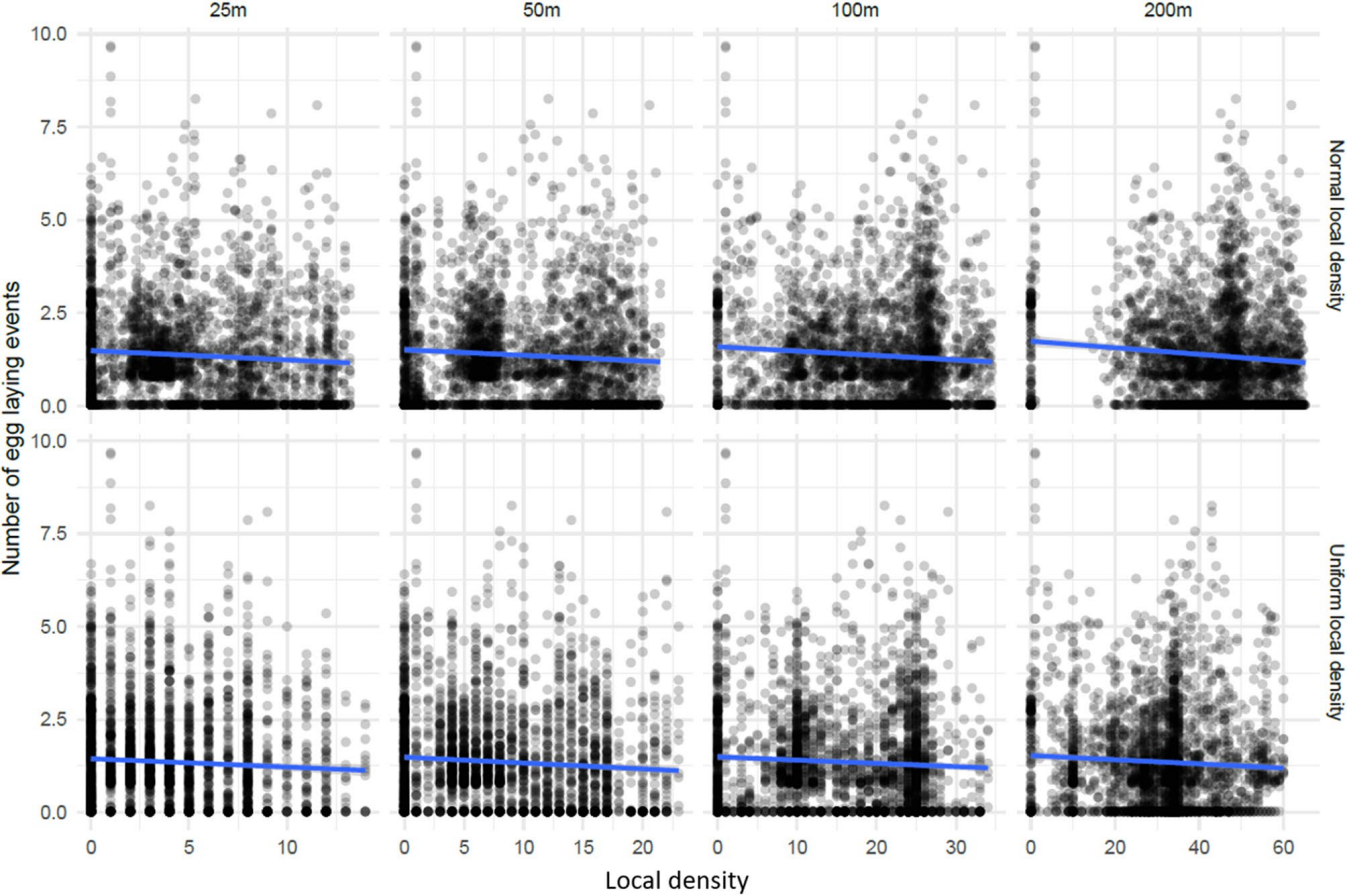
Number of inferred egg laying events and the local density of ovitraps in the previous week. This plot shows the number of inferred egg-laying events by the local density of ovitraps in the previous week for all ovitraps. The rows correspond to the Normal and Uniform local density models and the columns give the distance parameter for those models. The x-axis can be loosely interpreted as the number of other ovitraps that are within about the specified number of meters of the given ovitrap. The blue line shows the predicted mean of the linear model of the data.

Figure 6 shows fits of a simple linear model for eight different definitions of the local density of ovitraps (details in “Methods”). In all eight cases (four parameter values for each model), the p-value for the test $\beta =0$ (i.e. no effect of local density) was found to be less than 0.01 and in all cases the sign of $\beta$ was negative suggesting a decrease in the number of egg laying events as the local density of traps increases however the local density is measured. However, the magnitude of the effect was very small ranging from −0.024 to −0.008. For example, in the case of the uniform local density model with parameter 25 m ($\beta$=−0.022, p = 0.002), the predicted effect of adding 10 ovitraps in a 25 m radius of the index trap would, on average, reduce the number of egg laying events per week by about 0.2 or about 10 eggs per week in the index trap. Given the slight degree of heteroscedasticity in the data we also computed the p-values using robust random errors^35^, finding that the reported p-values were robust to the empirical degree of heteroscedasticity.

**Figure 7:**
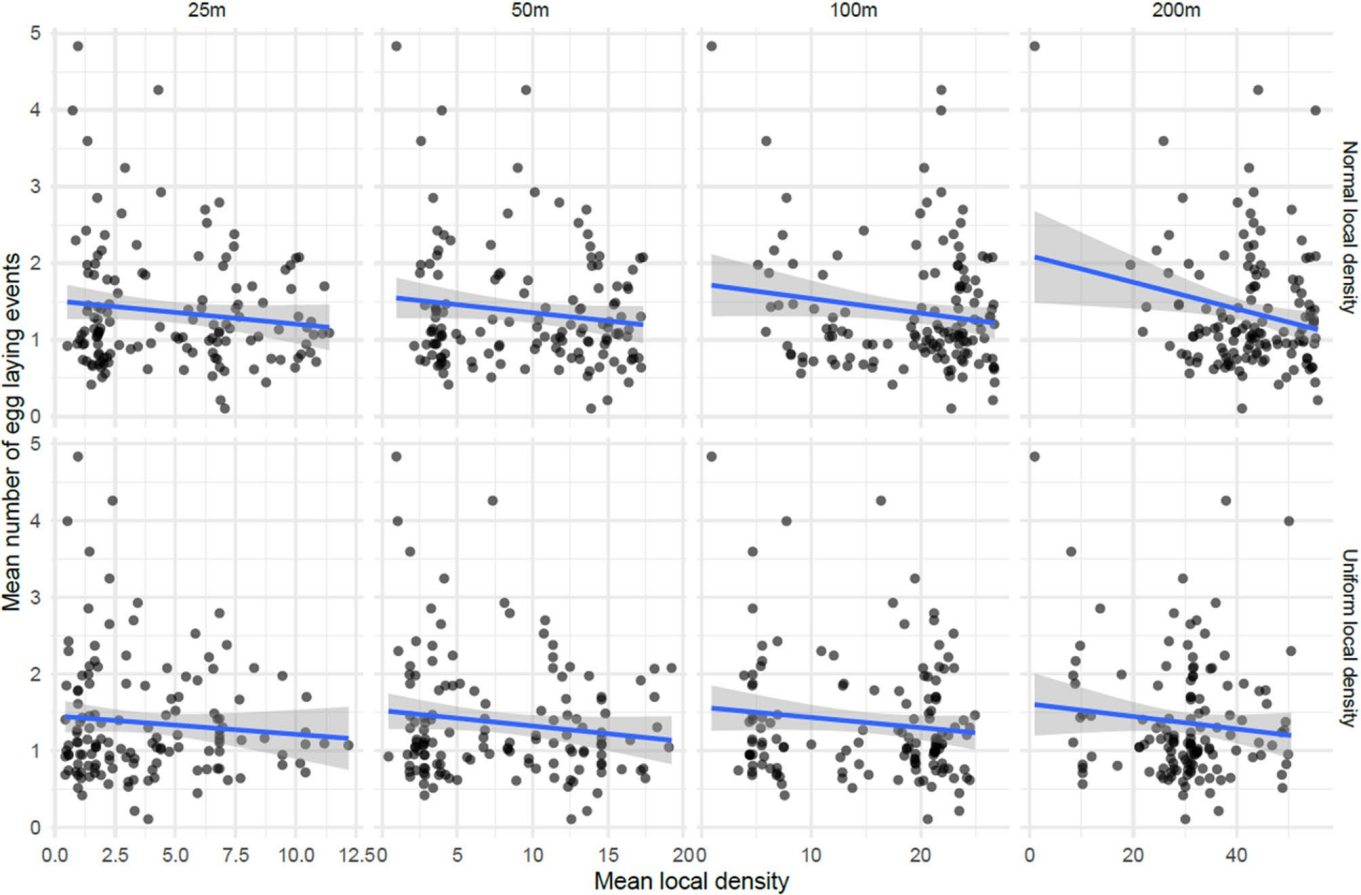
Mean Number of inferred egg laying events and the mean local density of ovitraps in the previous week over the course of the study. This plot shows the mean number of inferred egg laying events by the mean local density of ovitraps in the previous week for all ovitraps. The rows correspond to the Normal and Uniform local density models and the columns give the distance parameter for those models. The x-axis can be loosely interpreted as the number of other ovitraps that are within about the specified number of meters of the given ovitrap. The blue line shows the predicted mean of the linear model of the data.

We also considered the data and local density measures in aggregate over the course of the whole study. This allowed us to relax the one-week lag assumption in the previous model, which also has the advantage of averaging over secular trends in the mosquito population levels at the cost of a much smaller dataset. Results of those model fits are shown in Fig. 6. As expected with the much smaller datasets obtained by averaging over the course of the study, the measured p-values were much larger with only the Normal local density model with parameter 200 m having a p-value less than 0.05. In general, the effect measures were similar in the weekly model and the aggregated model (range −0.008 to −0.03). However, the general trend towards small but measurable reductions in the number of egg laying events with increasing local density of other ovitraps is more apparent in the aggregated data (Fig. 7).

## Discussion

In this investigation, we sought to obtain baseline information on the logistics associated with efforts directed toward large-scale deployment of yeast interfering RNA lure-and-kill ovitraps in a residential neighborhood in Curepe, Trinidad, West Indies. In a previous study conducted on the University of the West Indies campus, adjacent to Curepe, we determined that large 10 L ovitrap containers baited with yeast interfering RNA tablets were significantly more attractive than identical but unbaited containers to gravid *A. aegypti* and *A. albopictus* females^34^. As a proxy for simulating 100% lethality, our ovitraps were lined with seed germination papers that were removed and replaced weekly throughout the rainy season. Eggs were then counted to assess attractiveness of individual traps to gravid *Aedes* females and thereby also provide information on the anticipated number of larvae expected per ovitrap, particularly maximum larval numbers that our large volume lethal ovitraps will need to accommodate.

In general, the rainy season oviposition patterns in 10 L ovitraps distributed in the Curepe neighborhood were similar to those observed in a previous study on the adjacent University of the West Indies campus, with the greatest average egg numbers per trap occurring during August^34^. This pattern is also consistent with that observed for St. Joseph, Trinidad in 1988 using 400 ml ovitraps^23,24^. However, the maximum number of eggs collected in a single 10 L ovitrap during the current study was nearly double (1251 vs. 645) what we observed in the previous study, indicating that maximum oviposition rates in these traps are extremely variable and that effective yeast interfering RNA larvicide formulations need to consider this. As observed in Hapairai et al.^34^, both *A. aegypti* and *A. albopictus* were represented in ovitraps throughout the rainy season, although *A. aegypti* was predominant throughout the current study. This likely reflects the more urban environment in Curepe where the abundance of manmade containers is more conducive to *A. aegypti*, whereas the University of the West Indies campus likely offered fewer manmade containers and more natural breeding sites that are more conducive to *A. albopictus*^36,37^.

As the maximum number of eggs laid in individual traps was frequently over 400, it is also clear that multiple *Aedes* females were ovipositing in these traps. Previous molecular genetic analyses have confirmed that an average of 4.7 to 6.2 families were generally represented in individual containers, apparently independent of container size^18,38^, with up to nine families represented in a single container^18^. In addition, it is likely that individual females deposited eggs across multiple containers, a well-known behavior termed ‘skip-oviposition’^18,19,38–40^. Further, molecular analyses have also shown that individual females distributed eggs in containers separated up to at least 100 m^18^.

The observed small effect of higher-levels of ovitraps being near an index case is consistent with the reasonable assumption that removing eggs from circulation has an effect of reducing the local population of egg-laying adults. However, at the ovitrap densities obtained in this study the effects were statistically negligible. Of note too, median egg counts for individual ovitraps across the rainy season indicated that certain traps consistently attracted gravid *Aedes* females (Fig. 3). This phenomenon has previously been reported from other studies^41,42^, with the suggestion that microhabitat conditions around individual ovitraps likely plays an important role in egg laying behavior. However, Barrera et al.^42^ further showed that ovitrap attractiveness was mainly correlated with presence and attractiveness of nearby traps, rather than local microhabitat factors, including variables such as water volume, water temperatures, and trap exposure to rain and sun conditions. Thus, it has been concluded that microhabitat conditions play a less important role than deploying sufficient ovitraps to provide adequate coverage of neighborhoods^28,42^. Again, this may relate to the propensity of gravid *Aedes* females to exhibit skip oviposition among containers within a given area^18,19,38–40^.

Our results here and previously^34^ provide consistent evidence that our yeast-baited 10 L ovitraps are highly attractive to gravid *Aedes* females in an urban field setting. They also suggest that larger volume ovitraps may offer a better alternative for assessment and subsequent management of mosquito populations, including larviciding. That is, general surveillance programs have historically been based on monitoring oviposition activity using small (~ 500 ml) containers that, while readily attractive to gravid females, consistently collect fewer eggs than we have observed with larger volume containers. For example, previous studies in Trinidad with small volume ovitraps most frequently collected less than 60 eggs per week with a maximum of ~ 75 eggs^23,24^. This general pattern has remained consistent across multiple studies in various geographic regions that deployed similar-sized ovitraps^43–48^.

Consistent with other studies^46,49–51^, our results clearly highlight the need to include efforts directed at community participation and integrated control activities across neighborhoods. A primary difficulty we encountered in this study was in gaining access to residential properties in our efforts to saturate neighborhoods with ovitraps, even with direct assistance by staff of the IVCD. In most cases, the limitation was a consequence of the residents all likely being away for employment during the work day, and these residential compounds were typically protected by security fencing. The effects of this are evident in Fig. 2, where major areas of each Treatment block were inaccessible for ovitrap placement. This highlights the need to consider property inaccessibility in planning for future studies and how best to develop strategies that at a minimum would include efforts directed at more dense trap placements around the perimeters of these properties. We did simultaneously conduct several community engagement activities across Trinidad, including the Curepe area, prior to this study^52,53^, wherein we found that most residents were knowledgeable about mosquito biology and disease transmission, and also supportive of our yeast interfering RNA larvicides and their use in lethal ovitraps. Thus, it seems likely that including expanded community engagement activities prior to, and during future ovitrap trials, could promote increased neighbor-to-neighbor interactions that would facilitate access to and placement of ovitraps more effectively.

## Methods

### Study site

Field studies were conducted across sites in Curepe, Trinidad, West Indies (10^o^37′59.99″ N–61^o^23′59.99″ W) from June 26 until December 4, 2019. The town is located in the Tunapuna-Piarco Regional Corporation of Trinidad, covers ~ 4.34 km^2^, and is largely suburban with single and multi-family housing units mixed with small businesses, and a population of ~ 8,700 people. The study area encompassed an ~ 1.32 km^2^ portion of Curepe that is bordered by four major roads: the Priority Bus Route to the north, University Drive to the east, Churchill Roosevelt Highway to the south, and the Southern Main Road to the west (Fig. 1). The dry season typically occurs during December to May, and the wet season during May to November^54^.

### Study design

To gain preliminary information on the logistics associated with yeast-baited lethal ovitrap placement, effects of simulated 100% larval mortalities in our yeast-baited ovitraps, as well as rainy season oviposition activity in these traps by *Aedes* females, we selected three discrete block areas defined as ‘Treatment’ within the Curepe area (Fig. 1). With collaboration of staff from the Insect Vector Control Division (IVCD) at the Ministry of Health (MoH), the Treatment blocks were surveyed for: total number of buildings and building use, total number of people living in occupied buildings, and total number of potential *Aedes* breeding containers and container types irrespective of whether they contained water at the time or not. Based on these data, Treatment blocks were chosen based on human population densities (e.g., primarily residential vs. commercial buildings) and physical isolation from each other. Thereafter, we sought to place inactive yeast-baited ovitraps in areas outside occupied buildings. For the first three weeks, 10 traps were distributed across each Treatment block to obtain baseline data on oviposition activity. During weeks four and five, our goal was to eventually place individual ovitraps within 20–25 m of each other throughout these outdoor areas, such that gravid *Aedes* females would have ready access to one for oviposition within easy flight range. This distribution plan was based on mark-release-recapture studies conducted under similar environmental conditions in Florida and Puerto Rico, wherein results showed that gravid *Aedes* females dispersal distances are inversely correlated with ready access to oviposition sites, and that they typically travel less than 50 m from release sites if oviposition sites are available^55,56^. Ovitrap positions were selected to provide protection from wind, rainfall, and direct sunlight.

To gain information on background oviposition activity throughout the Curepe study area, we selected three additional blocks each as: (1) areas defined as ‘Control’ where no active *Aedes* control measures were implemented, and (2) areas defined at ‘Vector’ where personnel from IVCD performed periodic surveillance that involved identification of and either emptying or chemical treatment of potential breeding containers. These blocks were selected such that a Control and Vector was located within the adjacent areas of each Treatment block. In these areas, we distributed 10 yeast-baited ovitraps to obtain background information on oviposition rates throughout the rainy season, but not remove enough eggs to significantly impact the local mosquito populations.

Heat-inactivated yeast tablets were prepared using a transiently transformed *Saccharomyces cerevisiae* control construct^30^ as previously described^57^. A single ~ 70 mg tablet was placed in each ovitrap. This construct expresses a short hairpin RNA (shRNA) that has no identified target in mosquitoes or any other organism. This construct was used because our yeast-baited large ovitraps are highly attractive to gravid females,^34^ and the experiment design was not intended to directly test for larvicidal activity. Individual ovitraps in Treatment areas were serviced weekly, while those in the Control and Vector areas were serviced every two weeks and staggered such that servicing of the two types was performed on alternate weeks. Dark blue 10 L plastic buckets were used as ovitraps and were set up with ~ 3.5 L of tap water, the perimeter lined with seed germination paper (Anchor Paper Company, Saint Paul, MN, USA) as an oviposition substrate (egg paper), and covered with a 10 mm mesh wire screen. Detailed ovitrap descriptions were provided elsewhere^34^. During weekly servicing of individual ovitraps, egg papers were removed, ovitraps emptied and thoroughly rinsed with fresh tap water, refilled and provided with a clean egg paper and a new yeast tablet. Following collection, egg papers were transported to an insectary at the University of the West Indies, where they were allowed to dry at room temperature, and then total *Aedes* egg counts were performed for each. As both *A. aegypti* and *A. albopictus* are present in Trinidad^34,58^, we periodically hatched, reared, and identified adults to species from pooled samples of individual egg papers following our standard conditions^59^.

### Data analysis

#### Number of egg laying events

The total number of eggs laid in an ovitrap each week was expected to be highly variable based on our previous study^34^. High variability in signal can lead to spurious results, although this is mitigated by the long duration of the study. Because we want to measure the effect of local ovitrap density on the unobserved number of egg laying events rather than the raw counts of eggs in each trap, which are highly stochastic due to a variable number of eggs being laid in each laying event^40^, we inferred the number of egg laying events based on the observed number of laid eggs in each trap per week. This has the joint advantages of both naturally reducing the potential influence of outliers in the data—therefore reducing the likelihood of false positive results—and imputing missing values in the data. The model assumes the number of observed eggs in a given trap-week, ${y}_{i,t}$, is distributed as a Negative Binomial random variable conditional on the inferred number of egg laying events, ${y}_{i,t}^{{\prime}}$: ${y}_{i,t}\sim {\text{NegBin}}\left(\mu ={50y}_{i,t}^{{\prime}}, \sigma =10\right)$. Further, the model assumes a simple autoregressive process of the form ${y}_{i,t}^{{\prime}}={y}_{i,t-1}^{{\prime}}+ {\text{Normal}}\left(\mu =0, \sigma =\epsilon \right)$. The R library rstan 2.21.1^60^ was used to estimate the number of unobserved egg laying events and the variance of the Normal autogressive process, $\epsilon$. This is the same methodology as used in Haipairai et al.^34^.

#### Local density

We defined the local density of an ovitrap as a function of the number of other ovitraps within a given radius that were measured in the previous week. That is local density of ovitrap $i$ at time $t$ was calculated as

$${\omega }_{i,t}(\theta )=\sum_{A}{q}_{a}(t-1){f}_{\theta }(d(i,a))$$

where *A* is the set of all ovitraps, *q*_*a*_ is an indicator function that takes value 1 or 0 if ovitrap *a* was measured at time *t* − 1 or not respectively, and ${f}_{\theta }(d\left(i,a\right))$ is a function with parameter $\theta$ of the Euclidian distance between a pair of ovitraps. We considered two definitions of local density by choosing different ${f}_{\theta }$. For the measure “local density, uniform”, ${f}_{\theta }$ is an indicator function that takes value 1 if the distance between the two ovitraps is less than $\theta$ and for “local density, normal” ${f}_{\theta }$ is the normal density of $d\left(i,a\right)$ with standard deviation $\theta$. Note that to make the uniform and normal measures more comparable we regularized the normal density such that the maximum value was always equal to 1, therefore both of these measures have the loose interpretation of being “how many ovitraps are nearby”. We considered the following values of $\theta$: 25, 50, 100, and 200 m. Because there is temporal variance in the frequency in which traps were measured, the local density of an ovitrap can vary from week to week.

#### Statistical model

We considered two statistical models for the effect of local density on the number of egg laying events in a given week. The first model assumes that the effect of the local density of oviraps on the number of egg laying events has a simple linear form, ${y}_{i,t}^{{\prime}}\sim \alpha +\beta {\omega }_{i,t-1}(\theta )+{\text{Normal}}\left(\mu =0, \sigma =\epsilon \right)$, where the parameter $\beta$ is the change in number of egg laying events per week associated with an increase in the local density of ovitraps and $\epsilon$ is the standard deviation of the error. The second model aggregates over the time series by modeling the average number of egg laying events with the average local density of ovitraps, ${\overline{y} }_{i}^{{\prime}}\sim \alpha +\beta {\overline{\omega }}_{i}(\theta )+{\text{Normal}}\left(\mu =0, \sigma =\epsilon \right)$ where ${\overline{y} }_{i}$ and ${\overline{\omega }}_{i}(\theta )$ are the average number of weekly egg laying events over the study period and the average local density of ovitraps in the previous week over the course of the study period respectively.

### Ethics approval and informed consent to participate

Approval of field studies in Trinidad, West Indies was given by the Southwest Regional Health Authority, a division of the Trinidad and Tobago Ministry of Health. Household consent was provided verbally during IVCD routine surveillance visits to participating households, during which time IVCD staff gained permission from residents for both staff and the research team members to access the residences. Research team members then explained the nature of the study to the homeowners, who were able to approve or reject ovitrap placement. Ovitraps were clearly marked with a label indicating that the devices were being used in conjunction with a research study, and which included the name and local contact information for Dr. A. Mohammed. This work was funded by U.S. Department of Defense Awards W81XWH-17-1-0294 to MDS and W81XWH-17-1-0295 to DWS.

## Supplementary Information

Supplementary Information 1.

Supplementary Information 2.

## Data Availability

All data generated or analyzed during this study are included in this article.
