# Supplementary material for: Field trials reveal the complexities of deploying and evaluating the impacts of yeast-baited ovitraps on Aedes mosquito densities in Trinidad, West Indies
Source: Sci Rep. 2022 Mar 8;12:4047. doi: 10.1038/s41598-022-07910-0 (PMC8904463; doi:10.1038/s41598-022-07910-0)
Supplement: Supplementary file 1 — Supplementary Information 1. [file 41598_2022_7910_MOESM1_ESM.pdf]

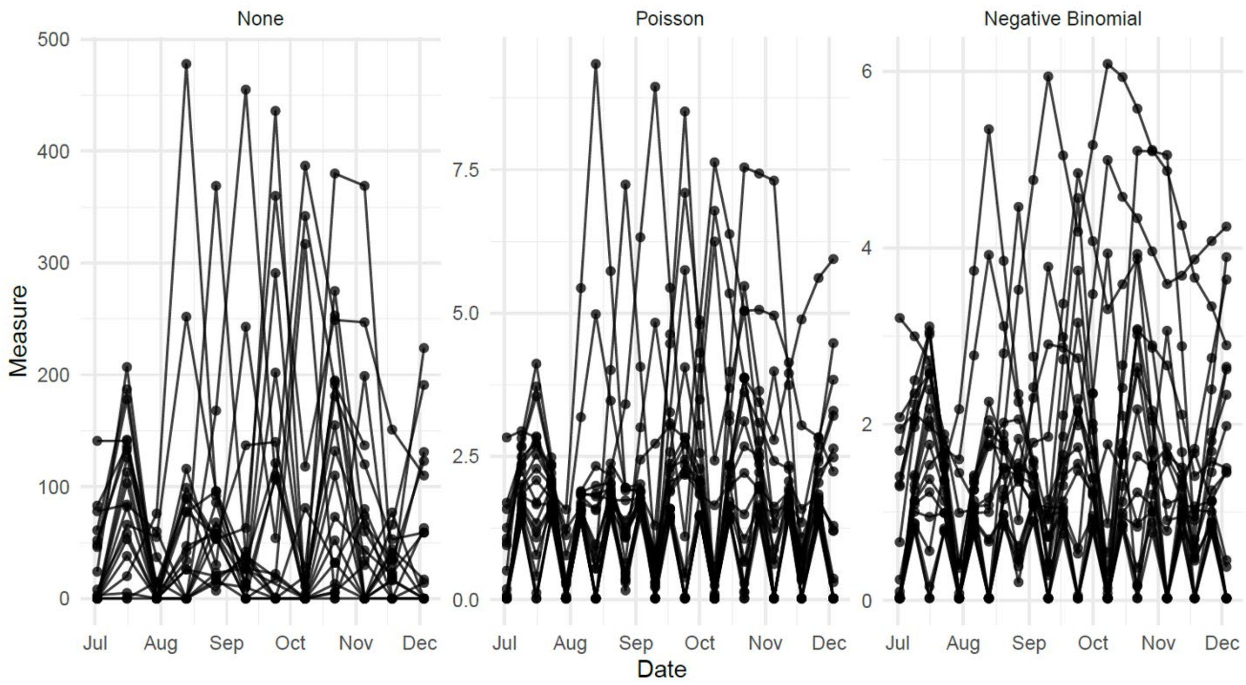

Supplementary Fig. S1. Inferred number of egg laying events under different models. The inferred number of egg laying events under a simple Poisson and Negative Binomial models are shown in addition to the raw data. Both models make different assumptions about the variance in the number of eggs laid in a single egg laying event with the Negative Binomial model allowing more heterogeneity in the number of eggs laid per event. The Negative Binomial model provides a greater level of smoothing and corrects for the effects of potential outliers on the downstream analyses.

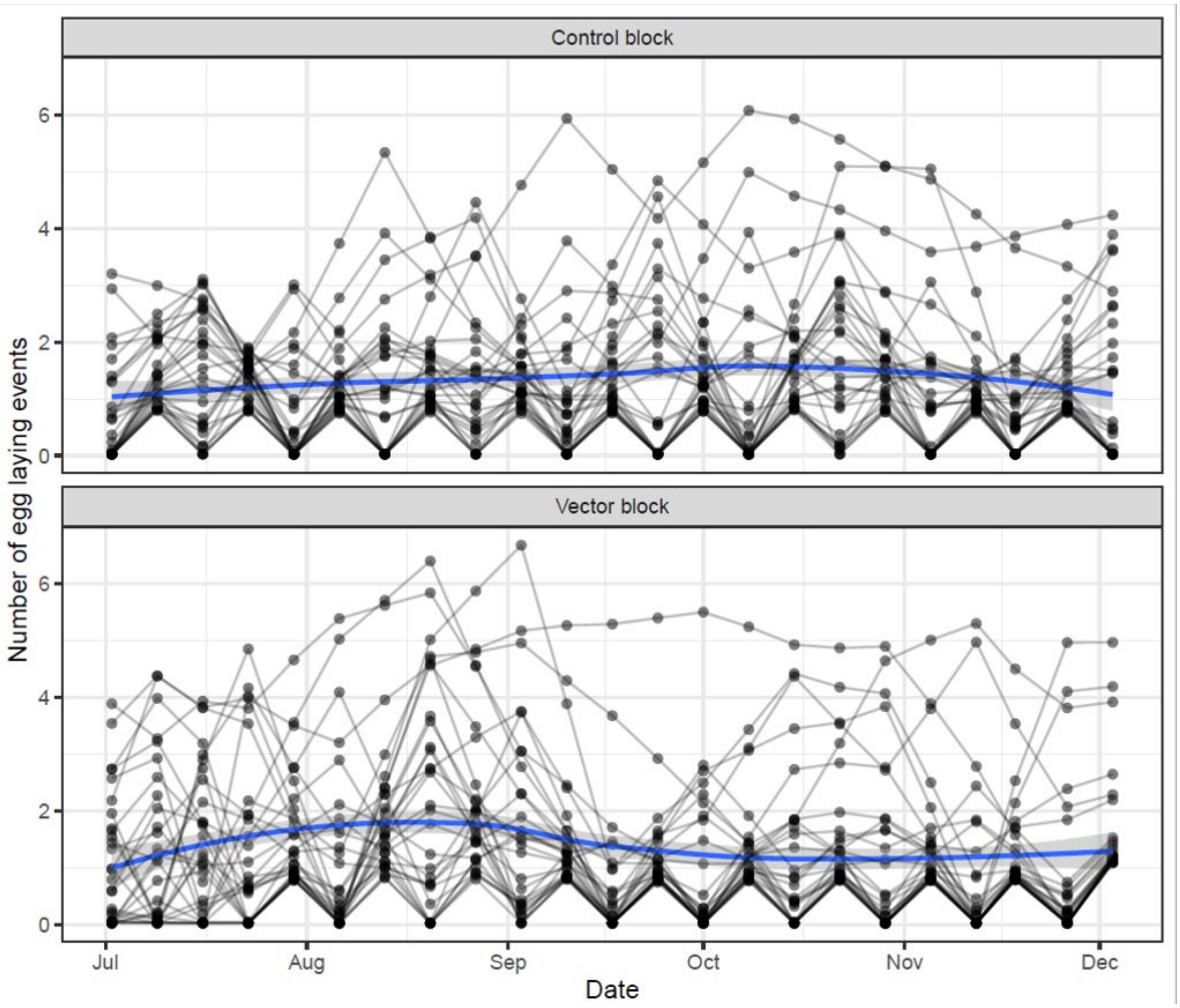

Supplementary Fig. S2. Number of egg-laying events in the Control and Vector blocks. The total number of inferred egg-laying events per week is plotted as a line for each ovitrap in the Control and Vector blocks. A loess regression line for each block is plotted in blue.
